# The heme sensing response regulator HssR in *Staphylococcus aureus *but not the homologous RR23 in *Listeria monocytogenes *modulates susceptibility to the antimicrobial peptide plectasin

**DOI:** 10.1186/1471-2180-10-307

**Published:** 2010-12-01

**Authors:** Line E Thomsen, Caroline T Gottlieb, Sanne Gottschalk, Tim T Wodskou, Hans-Henrik Kristensen, Lone Gram, Hanne Ingmer

**Affiliations:** 1 Department of Veterinary Disease Biology, University of Copenhagen, DK-1870 Frederiksberg C, Denmark; 2 National Institute of Aquatic Resources, Technical University of Denmark, DK-2800 Kgs. Lyngby; 3 NovoNordisk, DK-2880 Bagsvaerd, Denmark; 4 Novozymes A/S, DK-2880 Bagsvaerd, Denmark; 5 Chr. Hansen A/S, DK-2970 Hoersholm, Denmark

## Abstract

**Background:**

Host defence peptides (HDPs), also known as antimicrobial peptides (AMPs), have emerged as potential new therapeutics and their antimicrobial spectrum covers a wide range of target organisms. However, the mode of action and the genetics behind the bacterial response to HDPs is incompletely understood and such knowledge is required to evaluate their potential as antimicrobial therapeutics. Plectasin is a recently discovered HDP active against Gram-positive bacteria with the human pathogen, *Staphylococcus aureus *(*S. aureus*) being highly susceptible and the food borne pathogen, *Listeria monocytogenes *(*L. monocytogenes*) being less sensitive. In the present study we aimed to use transposon mutagenesis to determine the genetic basis for *S. aureus *and *L. monocytogenes *susceptibility to plectasin.

**Results:**

In order to identify genes that provide susceptibility to plectasin we constructed bacterial transposon mutant libraries of *S. aureus *NCTC8325-4 and *L. monocytogenes *4446 and screened for increased resistance to the peptide. No resistant mutants arose when *L. monocytogenes *was screened on plates containing 5 and 10 fold Minimal Inhibitory Concentration (MIC) of plectasin. However, in *S. aureus*, four mutants with insertion in the heme response regulator (*hssR*) were 2-4 fold more resistant to plectasin as compared to the wild type. The *hssR *mutation also enhanced resistance to the plectasin-like defensin eurocin, but not to other classes of HDPs or to other stressors tested. Addition of plectasin did not influence the expression of *hssR *or *hrtA*, a gene regulated by HssR. The genome of *L. monocytogenes *LO28 encodes a putative HssR homologue, RR23 (in *L. monocytogenes *EGD-e lmo2583) with 48% identity to the *S. aureus *HssR, but a mutation in the *rr23 *gene did not change the susceptibility of *L. monocytogenes *to plectasin.

**Conclusions:**

*S. aureus *HssR, but not the homologue RR23 from *L. monocytogenes*, provides susceptibility to the defensins plectasin and eurocin. Our data suggest that a functional difference between response regulators HssR and RR23 is responsible for the difference in plectasin susceptibility observed between *S. aureus *and *L. monocytogenes*.

## Background

Humans are living in a constant struggle with infectious microorganisms and whilst improved hygiene has been essential to control such organisms, one of the major steps forward has been the discovery and use of antibiotics. However, the high rate at which bacteria become resistant to currently used antibiotics is regarded as a major threat to the future treatment of infectious diseases in both humans and livestock [1,2]. Therefore, there is a growing demand for new types of antimicrobial compounds and interest is focused on host defence peptides (HDPs) as novel therapeutic agents. HDPs are a unique and diverse group of peptides, which can be grouped into different classes, based on their amino acid composition and structure. In humans and other mammals, the defensins and the cathelicidins constitute the two main HDP families. The cathelicidins vary widely in sequence, composition and structure, but share a highly conserved N-terminal structural domain (cathelin) linked to a highly variable cathelicidin peptide domain [3]. The defensins are more uniform, small cysteine-rich cationic peptides [4]. Defensins have well-established antimicrobial activity against a broad spectrum of pathogens, and in addition they have been shown to have immunostimulatory functions on both innate and adaptive immunity [5]. This has prompted a massive interest in synthetic defensins as novel antimicrobial candidates for therapeutic use.

Recently, the antimicrobial peptide, plectasin isolated from a saprophytic fungus, was described [6]. Plectasin is a defensin, which has broad activity against several species of Gram-positive bacteria and combined with very low toxicity in mice and on human keratinocytes and erythrocytes, plectasin holds promise as a novel anti-infective treatment [6,7].

In the present study, we addressed the response of two human pathogens, *S. aureus *and *L. monocytogenes *to plectasin. These two pathogens differ in sensitivity towards plectasin with MIC values of 16-32 mg/L for methicillin resistant *S. aureus *(MRSA), and above 64 mg/L for the less sensitive *L. monocytogenes *[6,7]. In addition, the two bacteria represent different routes of infection and may be exposed to different arrays of HDPs. *S. aureus *is a hospital- and community-acquired pathogen that causes a wide range of diseases including septicaemia, toxic-shock syndrome and food poisoning [8]. *S. aureus *is primarily extracellular and produces extracellular enzymes and toxins that cause damage to tissues. *L. monocytogenes *is a food borne pathogen causing gastroenteritis or septicaemia and meningitis in immunocompromised individuals [9]. As opposed to the infection mode of *S. aureus*, *L. monocytogenes *is an intracellular pathogen, able to spread from cell to cell within the host and thereby guarded against circulating immune factors.

The purpose of the present study was to investigate if resistance towards plectasin could be induced in *S. aureus *and *L. monocytogenes *by transposon mutagenesis and if this resistance would affect the mutants' response to other groups of antimicrobial peptides.

## Results

### Plectasin does not cause cellular leakage

**Figure 1:**
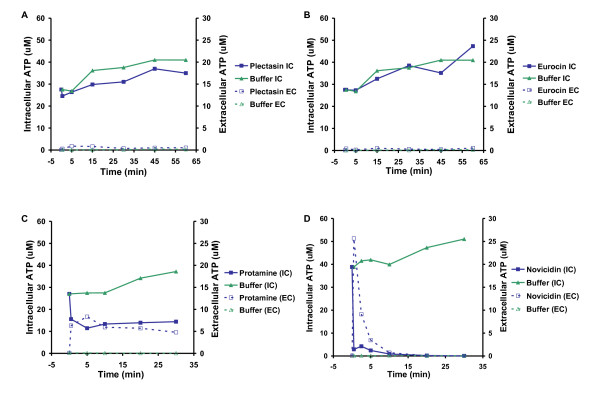
**Measurement of ATP leakage from *Staphylococcus aureus *after treatment with plectasin (A), eurocin (B), protamine (C), and novicidin (D)**. Measurement of intracellular (IC) and extracellular (EC) ATP after treatment with plectasin (500 μg/ml), eurocin (500 μg/ml), protamine (1,000 μg/ml), novicidin (1,000 μg/ml), or peptide dilution buffer. Treatment with the two defensins does not lead to leakage of intracellular ATP, whereas treatment with protamine and novicidin lead to leakage of ATP. Representative results from *S. aureus *are shown as treatment of *S. aureus *and *L. monocytogenes *resulted in similar leakage profiles. The experiment shown is representative of two independent experiments.

Many antimicrobial peptides affect the structural or functional integrity of the bacterial membrane, leading to pore formation and subsequently leakage of intracellular components [10]. Therefore, we examined the extracellular protein-profile by SDS-PAGE analysis. When the two Gram-positive pathogens, *S. aureus *and *L. monocytogenes*, were grown with and without plectasin, there was no difference, indicating that the bacteria are not leaking macromolecules (data not shown). To support this notion, we determined the effect of plectasin on the membrane of the two species by measuring the amount of ATP leakage. In this study we also included three peptides representing each of the antimicrobial peptide groups: the plectasin-like defensin eurocin, the linear arginine-rich peptide protamine and the α-helical peptide novicidin [11]. ATP leakage profiles were similar for *L. monocytogenes *and *S. aureus *but differed between peptides. When either of the pathogens was exposed to the defensins, plectasin or eurocin, we found that the intracellular ATP concentration remained at the same level as the controls treated with peptide dilution buffer only (Figure 1). This indicates that the defensins do not cause pore formation or membrane disruption in any of the bacteria. In contrast, protamine and novicidin resulted in increased ATP leakage thus suggesting that they are disrupting the membrane (Figure 1). Our finding is in agreement with recent results which revealed that plectasin targets the bacterial cell wall precursor Lipid II and does not compromise the membrane integrity [12].

Even though plectasin is known to interact with Lipid II, an increasing amount of evidence has established that HDPs can have multiple targets, including cytoplasmic and intracellular targets [13]. One such target is the bacterial DNA. However, we were unable to demonstrate binding of plectasin or eurocin to DNA when examined by *in vitro *gel retardation (data not shown).

### Identification of genes providing increased resistance to plectasin

In order to identify genes involved in the bacterial susceptibility towards plectasin, we created transposon mutant libraries in *S. aureus *8325-4 and *L. monocytogenes *4446 using *bursa aurealis *and Tn*917*, respectively. MIC values on agar plates were determined for the two wild types and the two transposon libraries were subsequently screened on plectasin-concentrations corresponding to 4, 5 or 10 fold MIC. After screening 40,000 colonies of *L. monocytogenes *transposon mutants, we found no mutants with increased resistance.

Screening of the *S. aureus *mutant library resulted in identification of four colonies with increased resistance, in which the transposon element had inserted into the heme response regulator *hssR *that together with *hssS *forms an operon, encoding a two component system (TCS) [14]. *S. aureus *require iron, and during infection, it can obtain iron through the haemolysin-mediated rupture of erythrocytes [15]. While heme is an important source of iron, high concentrations are toxic to *S. aureus *due to the reactivity of the molecule [16]. Therefore, the HssRS TCS is able to sense high concentrations of heme and induces the expression of *hrtAB*, encoding the HrtAB efflux pump that protects the cells against heme-mediated cell damage [16,17].

To control whether the selected plectasin concentrations induce spontaneous mutations, *S. aureus *and *L. monocytogenes *wild types were grown on TSB and BHI, respectively, with 4, 5 or 10 fold MIC. We found that no spontaneous mutations, leading to changes in sensitivity, occurred.

No mutants were obtained from the screening of the transposon mutant library of *L. monocytogenes *for altered resistance to plectasin. However, a homologous system in *L. monocytogenes *LO28 was identified by homology search and we found that the response regulator RR23 has a higher identity (48%) to HssR compared to other response regulators (30-35%) from *L. monocytogenes *LO28. In addition, RR23 show 99% amino acid sequence identity to *L. monocytogenes *EGD-e lmo2583, previously identified as an HssR homologue [14]. To evaluate the importance of HssR on sensitivity to plectasin of a bacterium other than *S. aureus*, a *L. monocytogenes rr23 *mutant was included in the experiments.

### HssR modulates resistance to defensins

In order to validate the phenotypes obtained by our *S. aureus *transposon mutant 8325-4 *hssR*::*bursa*, we transduced the transposon element from 8325-4 *hssR*::*bursa *to *S. aureus *8325-4 wild type, giving the mutant 8325-4 *hssR*. In addition, we included another *S. aureus *wild type, *S. aureus *15981, and the 15981-ΔTCS15 (15981-ΔTCS15 *hssRS*), which harbour a deletion of both the response regulator *hssR *and the histidine kinase *hssS *[18].

The MIC for plectasin was determined for all the strains using the microbroth dilution method (Table 1) and a mutation in the *hssR *response regulator in *S. aureus *lead to a 2 to 4 fold increased resistance compared to the wild type, regardless of the genetic background. This is in agreement with the initial finding, where we used 4 fold MIC in the plate screen for transposon mutants. A complementation of 8325-4 *hssR::bursa *(8325-4 *hssR::bursa*/pRMC2-*hssRS*) decreased the resistance 2 fold compared to the 8325-4 *hssR::bursa *(Table 1). The deletion of the *rr23 *in *L. monocytogenes *had no effect on the resistance towards plectasin (Table 1).

**Table 1:** MIC values of host defence peptides against *S. aureus *and *L. monocytogenes *wild types and two-component system mutants.

		MIC (μg/ml)				
		
Strain	Description	Plec	Euro	Prot	NovC	NovS
8325-4	*S. aureus *wild type	16	32	16	1	128
8325-4 *hssR::bursa*	Transposon mutant	32	64	16	1	128
8325-4 *hssR::bursa*/pRMC2-*hssRS*	Complementation of *hssR *transposon mutant	16	32	nd	nd	nd
8325-4 *hssR*	Transduced 8325-4 *hssR *mutant	32	64	16	1	128
15981	*S. aureus *wild type	8	8	16	1	>128
15981 *ΔTCS15 (hssRS)*	*hssRS *deletion mutant	32	32	16	1	>128
LO28	*L. monocytogenes *wild type	64	128	16	1	16
LO28 RR23	*rr23 *insertion mutant	64	128	16	1	16

Plec: plectasin, Euro: eurocin, Prot: protamine, NovC: novicidin, NovS: novispirin. nd: not determined.

In addition, we tested whether the two-component system is involved in altered sensitivity to other antimicrobial peptides namely novispirin (a cathelicidin), novicidin (a cathelicidin), protamine (a linear peptide) and eurocin (a plectasin-like defensin). The *S. aureus hssR*/*hssRS *mutants were also more resistant to eurocin, the only other defensin, but were not altered in sensitivity to other groups of peptides (Table 1).

**Figure 2:**
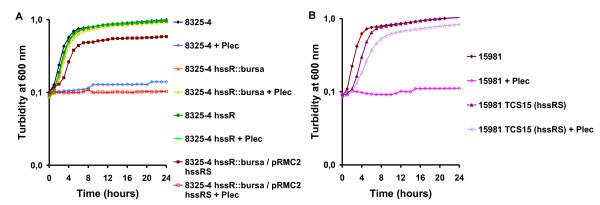
**Growth of *S. aureus *8325-4 (A) and 15981 (B) wild types and *hssR *mutants in the presence of plectasin**. Plectasin (35 μg/ml) inhibited the growth of *S. aureus *8325-4 and 15981 wild-types but hardly affected the growth of the 8325-4 *hssR*::*bursa *transposon mutant, the transduced 8235-4 *hssR *mutant or the 15981 ΔTCS15 (*hssRS*) mutant. Complementation of 8325-4 *hssR*::*bursa *(8325-4 *hssR*::*bursa*/pRMC2-*hssRS) *affected the growth slightly, but addition of plectasin inhibited the growth to a level comparable to wild type. The experiment shown is representative of three independent experiments.

**Figure 3:**
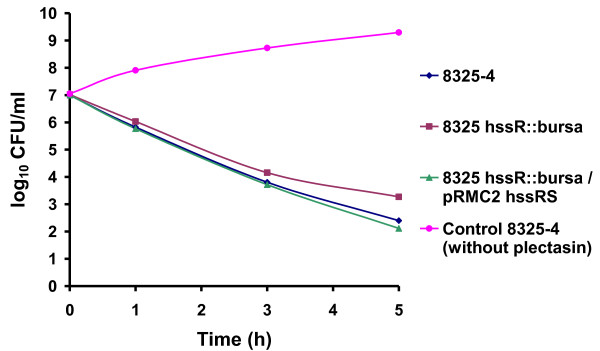
**Kinetics of bacterial killing *in vitro***. *S. aureus *8325-4 wild type, 8325-4 *hssR::bursa and *8325-4 *hssR::bursa*/p RMC2-*hssRS *were incubated in the presence of 1XMIC. The colony counts are shown as representative of three independent experiments. CFU, colony-forming units.

The ability of the *S. aureus hssR *mutants to cope with higher concentrations of the peptide compared to the wild type was confirmed in a growth experiment. The strains were grown with plectasin (in concentrations known to inhibit growth) or without plectasin. The wild type did not grow in the presence of plectasin, but the response regulator mutants all grew (Figure 2). Complementing 8325-4 *hssR::bursa *(8325-4 *hssR::bursa*/pRMC2-*hssRS*) lead to plectasin inhibited growth comparable to the growth of wild type (Figure 2A). The growth experiment also showed that the mutant and wild type strains have similar growth kinetics when grown in TSB (Figure 2). *In vitro*, *S. aureus *8325-4 was killed rapidly by plectasin (1× MIC), confirming the results from Mygind *et al *[6]. The 8325-4 *hssR::bursa *mutant was killed slower than the wild type (Figure 3).

**Figure 4:**
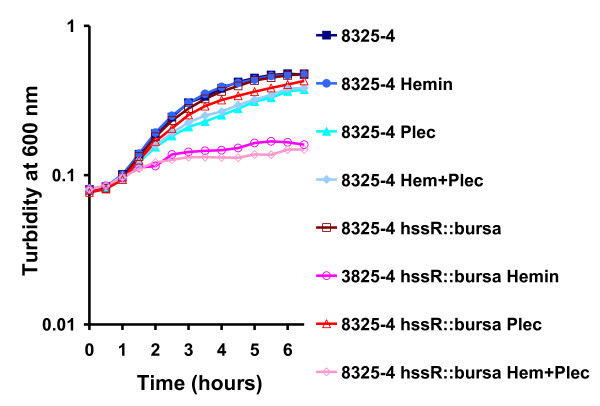
**Growth of *Staphylococcus aureus *wild type and *hssR *mutants in the presence of hemin and plectasin**. The growth of the *S. aureus *8325-4 wild type is only affected by plectasin (35 μg/ml) and not hemin (4 μM). On the contrary, the 8325-4 *hssR *mutants do not grow in the presence of hemin, regardless of the presence or absence of plectasin, confirming the heme-sensitive phenotype of *hssR *mutants. The experiment shown is representative of three independent experiments.

Both HrtAB and HssRS are required for growth of *S. aureus *in hemin [14]. When we examined the growth of the *hssR *mutant compared to the wild type we also found it to be almost completely inhibited by 4 μM hemin, regardless of the presence or absence of plectasin (Figure 4). The expression of *hrtAB *efflux system has previously been shown to increase 45 fold by exposure to hemin through transcriptional activation by HssR [19]. However, we found no change of expression of *hrtB *and *hssR *in the wild type when plectasin was added using northern blot and quantitative real-time PCR (P > 0.05).

### Plectasin does not affect protein secretion

Recent work has shown that exposing *hrtA *mutants to hemin, leads to increased protein secretion, however, when exposing *hssR *mutants to hemin, a similar change in secretion was not observed [14,20]. To investigate whether plectasin induces a change in protein secretion, we compared the *L. monocytogenes *and *S. aureus *wild types to the *hssR *mutants. We found no difference in the abundance of extracellular proteins, when the strains were grown with or without plectasin (data not shown).

### Stress and antibiotic resistance of *hssR *mutant cells

The relatively small number of TCSs in *S. aureus *and *L. monocytogenes *imply that some of them are able to sense several different stressors. In *Streptococcus pyogenes *the TCS CovRS, senses both iron starvation, antimicrobial peptides and several other stressors [21]. We have found that HssR affects the resistance towards defensins in addition to heme concentrations, we therefore determined if the HssRS TCS affects susceptibility to other types of stress. However, when the *S. aureus *and *L. monocytogenes *wild types and mutants were subjected to a variety of stress-conditions; growth at 15°C, 30°C, 37°C or 44°C, or growth with the addition of 4% NaCl, we found no difference in growth between the wild types and their respective mutants. We also examined the sensitivity of wild type and mutants to several antibiotics, i.e. ampicillin, gentamicin, sulfa/trimethoprim, rifampicin, tetracycline, amoxy/clavulan, cephalotin, clindamycin, enrofloxacin, fusidic acid and oxacillin. No change in MIC values was observed when the wild type *S. aureus *and *L. monocytogenes *and the corresponding response regulator mutants were compared (data not shown). Thus, as opposed to the CovRS TCS, HssR/RR23 from *S. aureus *and *L. monocytogenes *do not seem to sense other types of stress. The results for RR23 correspond with previous experiments, showing no stress phenotype for an *rr23 *mutant [22].

## Discussion

In the present study, we investigated how the antimicrobial peptide, plectasin, affects two human pathogens. Our results indicate that plectasin and another defensin, eurocin, do not perturb the *S. aureus *and *L. monocytogenes *membrane, but differentially affect the bacterial survival. These results are in agreement with recent findings, which show that plectasin does not compromise membrane integrity [6,12]. However, the non-defensins, novicidin and protamine did lead to increased leakage, implying that the antimicrobial activity of these peptides involves disruptions of the bacterial membranes (Figure 1).

To identify genes involved in resistance to plectasin, we screened transposon mutant libraries of *L. monocytogenes *and *S. aureus*. We were unable to identify any *L. monocytogenes *mutants more resistant to the peptide compared to wild type. The *L. monocytogenes *wild-type is more tolerant to plectasin (MIC >64 μg/ml) compared to the *S. aureus *wild type (MIC = 8-16 μg/ml), which might explain the difficulties in obtaining *L. monocytogenes *mutants with decreased sensitivity [[6,7], this work]. Four isolated *S. aureus *mutants, more resistant to plectasin, had the transposon element inserted in the response regulator *hssR *that is part of a TCS, HssRS, involved in sensing heme concentrations [14]. A primary mechanism by which bacterial cells respond to changes in the environment is through the action of TCSs. TCSs typically consist of a membrane-bound histidine kinase that responds to environmental signals by undergoing autophosphorylation followed by transfer of the phosphoryl group to the regulator [23]. During contact with a host, *S. aureus *acquire heme as iron source, but surplus heme can be toxic. The HssRS system is important for sensing the level of heme, and for activating the ABC transporter system HrtAB, which protects the bacteria against heme-mediated damage [16,17]. Changes in iron availability are an environmental signal indicative of mammalian host-pathogen interaction and the HssRS TCS seems to be important for *S. aureus *to sense and respond to heme as a component of vertebrate blood [24,14].

Our results reveal that a mutation in *hssR *increases the resistance of *S. aureus *to two defensin-like HDPs, suggesting that the mutation of *hssR *leads to enhanced bacterial resistance to immune clearance. Defensins are an important part of the mammalian immune response and mutant pathogens, more susceptible to HDPs, are often attenuated in virulence, indicating that the intrinsic resistance to HDPs plays a key role in bacterial infection [25]. Recently, a link between iron starvation and HDP resistance in *Yersinia pseudotuberculosis *has been shown, supporting the idea that bacteria can sense when inside a host and coordinate their response accordingly [26].

It has previously been reported that a mutation in *S. aureus hssR *or *hrtA *leads to increased virulence [14]. This increase has been suggested to be due to pore formation in the bacterial cell membrane that elicits an increased secretion of immunomodulatory factors, which decreases killing of the bacteria [20]. However, plectasin does not form pores or leads to increased secretion of bacterial compounds. These results indicate that the deletion of *hssR *affects the bacteria in a way that improve their ability to survive defensins of the host defence system causing the observed hypervirulence [14].

We did not observe an upregulation of *hssR *or *hrtB *when *S. aureus *was exposed to plectasin. Previous results have shown a 45 fold upregulation of *hrtAB *when exposed to exogenous hemin [19]. The lack of plectasin regulation of the systems implies that the TCS does not sense the defensins and the ABC transporter system HrtAB is not involved in exporting the peptides. This suggests that the lack of *hssR *alleviates a regulation of one or more target genes leading to the resistant phenotype. Modifications of the cell surface are known to affect HDP resistance and plectasin targets bacterial cell wall precursor Lipid II, implying a function of HssR on the cell wall synthesis or composition. Change in surface charge is known to affect HDP susceptibility and we have previously shown that *mprF *and *dltA *mutations affect *S. aureus *sensitivity to plectasin, novicidin, protamine and novispirin [7]. However, the effect of the *hssR *mutation is probably not due to changes in surface charge, since only plectasin and eurocin susceptibilities are altered.

A consensus DNA binding sequence for HssR has been predicted and genomic analysis of *S. aureus *has revealed that, besides the consensus sequence in the *hrtAB *promoter region, 14 genes have consensus sites containing 3-4 mismatches [16]. Whether one of these genes is involved in the observed plectasin resistance remains elusive. Further work is needed to clarify whether the *hssR *mutation has an effect on one of these genes in order to understand the bacterial changes that lead to reduced plectasin sensitivity.

Homologues of the HrtAB and HssRS systems are found in several Gram-positive bacterial pathogens [[14], this work]. A possible HssR homologue, RR23, exists in *L. monocytogenes*. However, a mutation in this response regulator did not affect growth or survival when exposed to the peptides and previous results have shown that RR23 is not important for virulence [22]. Our results show that the susceptibility to defensins can be decreased in *S. aureus *but not in *L. monocytogenes*. This inability to obtain more resistant *L. monocytogenes *mutants could be explained by the difference in MIC values between the strains, showing that *L. monocytogenes *is 4-8 fold more tolerant to plectasin compared to *S. aureus*. Whether this difference in sensitivity towards plectasin between *L. monocytogenes *and *S. aureus *can be explained by the variations in virulence factors and different routes of infection of the two pathogens remains elusive.

## Conclusions

We found that the *S. aureus *response regulator HssR, but not the corresponding RR23 from *L. monocytogenes*, is involved in the organisms' sensitivity to defensins, exemplified by plectasin. The mutation of *hssR *leads to increased resistance towards plectasin and eurocin. The HssRS two component system have previously been shown to be important for heme homeostasis and an *hssR *mutation leads to increased virulence [14]. Taken together these results further indicate the importance of this system in sensing environmental cues and responding accordingly. This result support the notion that the system is able to sense internal host tissue and shift to an immune evasive response and that the mutation in *hssR *leads to enhanced bacterial resistance to host immune factors. During the course of infection, the bacteria must not only cope with iron starvation but also resist antimicrobial peptides, including defensins. Whether the difference in responding to the HDPs between *L. monocytogenes *and *S. aureus *is due to the differences in infection processes still remains unclear. However, our results indicate a functional difference between RR23 and HssR and the genes regulated by these regulators, which might explain the difference in HDP susceptibility between the two strains.

## Methods

### Strains, plasmids and culture conditions

Bacterial strains and plasmids are described in Table 2. For complementation, a PCR amplification of *hssRS *was cut (KpnI-SacI) and cloned into the KpnI-SacI sites of pRMC2, transformed into *E. coli *DH5α (Invitrogen) and further transformed into 8325-4 *hssR::bursa*. Primers for amplifying *hssRS*: Complement1-Forward-KpnI:(5'ATCAGGGTACCGAAAAAGATAAGGGAGTTTA3'), Complement3-Reverse-SacI:(5'CGCTGAGCTCTTTCAGGAGGTAGAGATTAA3'). The 8325-4 *hssR *insertion mutant was constructed by φ11-mediated generalized transduction as previously described [27].

**Table 2:** Strains and plasmids used in this study

Strains	Relevant characteristic	Reference
*S. aureus *8325-4	wild type	[27]
8325-4 *hssR*::*bursa*	resistant mutant, *bursa *insertion	This work
8325-4 *hssR*	*hssR *mutation transduced from 8325-4 *hssR*::*bursa*	This work
*S. aureus *15981	wild type	[34]
*S. aureus *15981ΔTCS15	*hssRS *deletion	[18]
8325-4 *hssR*::*bursa*/pRMC2-*hssRS*	Complementation of the transposon mutant	This work
*L. monocytogenes *4446	wild type	[35]
*L. monocytogenes *LO28	wild type	[36]
LO28 RR23	rr23 mutant	[22]
**Plasmids**		
pRMC2	Tetracycline inducible expression vector	[37]
pRMC2-*hssRS*	*hssRS *cloned into pRMC2, complementation plasmid	This work

The bacteria were grown in Brain Heart Infusion broth (BHI, CM0225 Oxoid) (*L. monocytogenes*) or Tryptone Soy Broth (TSB, CM0129 Oxoid) (*S. aureus*). When appropriate, antibiotics were added at the following concentrations erythromycin 5 μg/ml (*L. monocytogenes*) and 10 μg/ml (*S. aureus*), chloramphenicol 10 μg/ml, tetracycline 12.5 μg/ml (Sigma) and 200 ng/ml anhydrotetracycline (Sigma).

### Host defence peptides

Protamine was purchased from Sigma (P4020-5G). Plectasin, eurocin, novicidin, and novispirin G10 were supplied by Department of Antiinfective Discovery, Novozymes A/S. The host defence peptides were dissolved in 0.01% acetic acid/0.1% bovine serum albumin (Sigma, A7906).

### Determination of the effect of plectasin on the bacterial envelope - ATP measurements

*L. monocytogenes *and *S. aureus *were grown in TSB at 37°C. Bacteria were harvested (10 min at 3000 RPM) at mid-exponential phase (absorbance at 546 nm of 2.5 ± 0.2 and 1.0 ± 0.2 for *S. aureus *and *L. monocytogenes*, respectively), washed once in 50 mM potassium phosphate buffer pH 7.0 and once in 50 mM HEPES buffer pH 7.0. The pellet was resuspended in 50 mM HEPES pH 7.0 to a final absorbance at 546 nm of 10. Bacteria were stored on ice and used within 5 hours. Bacteria were energized in 50 mM HEPES (pH 7.0) with 0.2% (wt/vol) glucose and treated with 500 μg/ml plectasin or eurocin. ATP was determined using a bioluminescence kit (Sigma, FLAA-1KT) and a BioOrbit 1253 luminometer. Total ATP content was determined by rapidly permeabilising 20 μl cell suspension with 80 μl dimethyl sulfoxide. The cell suspension was diluted in 4.9 ml sterile water, and ATP content was determined in 100 μl of the preparation as described by the manufacturer. To determine the extracellular ATP concentration, the 20 μl cell suspension was mixed with 80 μl sterile water and analyzed as described above. Intracellular ATP concentrations were calculated by using the intracellular volumes of 0.85 and 1.7 μm^3 ^for *S. aureus *and *L. monocytogenes*, respectively. The number of cells in suspension was determined by plate spreading.

### Extracellular protein

Prewarmed TSB and BHI (25 ml) in a 250 ml Erlenmeyer flask was inoculated with *S. aureus *strains and *L. monocytogenes *strains, respectively. These flasks were grown with and without plectasin at 37°C overnight (≈ 17 h) with shaking. The next morning, the exact absorbance at 600 nm of the cultures was measured, and 15 ml of culture was centrifuged to precipitate the cells (6 000 RPM; 7 min; 0°C). The supernatant was transferred to a 50 ml Blue cap bottle (placed in an ice/water bath), and the extracellular proteins were precipitated by adding one volume of ice-cold 96% EtOH and left in the refrigerator overnight for proteins to precipitate. Precipitated proteins were collected by centrifugation (11,000 RPM; 30 min; 0°C). Protein pellets were suspended in a volume of 50 mM Tris-HCl (pH 6.8) adjusted to the original absorbance of the overnight culture so that 15 ml of overnight culture with absorbance at 600 nm of 5.0 was suspended in 0.8 ml of 50 mM Tris-HCl (pH 6.8). A sample of 15 μl of the protein extracts was analysed on NuPAGE^® ^4-12% Bis-Tris gels (Invitrogen) using the X Cell SureLock^® ^Mini-Cell system (Invitrogen) as recommended by the supplier. The gels were Coomassie stained using GelCode^® ^Blue Stain Reagent (Pierce).

### DNA-binding analysis

Gel retardation analysis were performed as described by Nan *et al *by mixing 100 ng of plasmid DNA (pBluescript II SK^+^(Stratagene)) with increasing amounts of peptide in 20 μl binding buffer (5% glycerol, 10 mM Tris, 1 mM EDTA, 1 mM dithiothreitol, 20 mM KCL and 50 μg ml^-1 ^bovine serum albumin) [28]. Reaction mixtures were incubated 1 h at room temperature and subjected to 1% agarose gel electrophoresis and visualised using ethidium bromide.

### Transposon library in *L. monocytogenes *and *S. aureus*

Transposon mutagenesis of *L. monocytogenes *4446 was performed with the temperature-sensitive plasmid pLTV1 as described, but with modifications [29]. *L. monocytogenes *4446 harbouring pLTV1 was grown overnight at 30°C in BHI containing 5 μg/ml erythromycin. The bacterial culture was then diluted 1:200 in BHI containing 5 μg/ml erythromycin and grown for 6 h at 42°C. Aliquots were plated onto BHI containing 5 μg/ml erythromycin plates and incubated at 42°C. Colonies were harvested from the plates in BHI and stored in 30% glycerol at -80°C. To determine the transposition frequency, the transposon library was plated onto BHI containing 5 μg/ml erythromycin. One hundred colonies were picked and streaked onto BHI plates containing 5 μg/ml erythromycin, 10 μg/ml chloramphenicol, and 12.5 μg/ml tetracycline, respectively, and incubated at 30°C for 48 h. The transposition frequency was calculated as the percentage of colonies growing only on BHI + 5 μg/ml erythromycin and BHI+10 μg/ml chloramphenicol (harbouring only the transposon) but not on BHI+12.5 μg/ml tetracycline (still harbouring the plasmid). Transposon mutagenesis of *S. aureus *8325-4 with *bursa aurealis *was performed as described [30].

### Screening of transposon library for plectasin resistant mutants

The transposon mutant libraries were screened on agar plates for increased resistance to plectasin as compared to wild-type sensitivity. Wild-type sensitivity was determined by plating approx. 1.0 × 10^7 ^CFU/ml on TSB agar containing plectasin (*S. aureus*) and approx. 1.0 × 10^5 ^CFU/ml on Muller Hinton Broth agar plates (MHB, 212322 Becton Dickinson) with plectasin (*L. monocytogenes*). Plates were incubated at 37°C for 3 days and inspected for growth. The transposon libraries were screened on TSB agar with 300 μg/ml, 500 or 750 μg/ml plectasin (*S. aureus*) or MHB plates with 250 μg/ml or 500 μg/ml plectasin (*L. monocytogenes*) at 37°C for up to 7 days.

### Identification of transposon mutant

Chromosomal DNA was purified from resistant mutants using FAST DNA kit, Bio101, Qiagen, Germany). Identification was performed as described by Bae *et al *[30]. AciI was used to digest chromosomal DNA for 3 h at 37°C and thereafter ligated with T4 ligase. The ligated DNA was purified with the QIAquick PCR purification kit (Qiagen, Germany). DNA fragments carrying transposon/chromosome junction sequences were amplified by PCR with the following primers: Martn-F (5' TTT ATG GTA CCA TTT CAT TTT CCT GCT TTT TC 3') and Martn-ermR (5'AAA CTG ATT TTT AGT AAA CAG TTG ACG ATA TTC 3'). The annealing temperature was 63°C, and the DNA was amplified for 3 min with 40 cycles. PCR products were TOPO cloned according to the manufacturer (Invitrogen, USA). Plasmids were sequenced using M13 forward (5'GTAAAACGACGGCCAGT 3') and M13 reverse (5'AACAGCTATGACCATG 3').

### Determination of Minimum Inhibitory Concentrations (MIC) of antimicrobial peptides in liquid medium

Minimal inhibitory concentrations (MIC) of plectasin, eurocin, protamine, novicidin, and novispirin G10 were determined using a microbroth dilution method [31]. Colonies from a BHI plate incubated overnight at 37ºC were suspended in MHB pH 7.4 to an absorbance at 546 nm of 0.11-0.12 at 546 nm (approx. 1.0 × 10^8 ^CFU/ml) and diluted in MHB to a concentration of 5.0 × 10^5 ^CFU/ml. Ninety μl of bacterial suspension was incubated with 10 μl of peptide solution in polypropylene 96-well plates (Nunc, 442587) for 18-24 h at 37°C. The peptide solutions were made fresh on the day of assay. The range of concentrations assayed were 0.25-256 μg/ml for plectasin and eurocin, 0.125-128 μg/ml for protamine and novispirin G10, and 0.031-32 μg/ml for novicidin. MIC was the lowest peptide concentration at which visual growth was inhibited.

### Influence of hemin and plectasin on growth of *S. aureus*

Overnight cultures of *S. aureus *were diluted to an absorbance at 600 nm of 0.05 in TSB with and without 4 μM hemin and/or 35 μg/ml plectasin and grown at 37°C. Measurements of the absorbance were made every 30 minutes.

### *In vitro *bacterial killing

Overnight cultures of *S. aureus *wild type 8435-4, 8325-4 *hssR::bursa *and 8325-4 *hssR::bursa*/pRMC2-*hssRS *were diluted 1000 fold in TSB and grown 2 hours at 37°C. Samples were taken to time T = 0 and plated for CFU determination. Plectasin (1× MIC) was added, and samples were withdrawn after 1,3 and 5 hours growth at 37°C and plated for CFU determination.

### Potential influence of plectasin on *hssR *and *hrtB *expression

Wild type *S. aureus *and the *hssR *mutant were grown to an absorbance at 600 nm of 0.45 ± 0.1, samples were withdrawn for the isolation of RNA. Plectasin (35 μg/ml) was added to the growing culture, and after 10 and 90 minutes samples were also withdrawn. Cells were quickly cooled and lysed mechanically using the FastPrep machine (Bio101; Q-biogene), and RNA was isolated by the RNeasy kit (QIAGEN, Valencia, Calif.) according to the manufacturer's instructions. Northern Blotting: RNA was transferred to a nylon membrane (Boehringer Mannheim) by capillary blotting as previously described [32,33]. Hybridization was performed using gene-specific probes that had been labelled with [^32^P]dCTP using the Ready-to-Go DNA-labelling beads from Amersham Biosciences. Primers for probes amplifying *hrtB *and *hssR*: hrtB-1F:(5'CACTCAATAAATGTCTTGTC3'), hrtB-2R: (5'AAGGTAATTCATCAAGAACC3'), hssR-1F: (5'AATGTCTTGTTGTCGATGAC3'), hssR-2R:(5' TTATAGCCTTGTCCTCTTAC3'). All steps were repeated in two independent experiments giving similar results. Quantitative RT-PCR: RNA was treated with DNase and RevertAid™ H Minus first strand cDNA synthesis Kit (Fermentas). The Mx30000P^® ^and Maxima^® ^SYBR Green/ROX qPCR Master Mix (Fermentas) was used essentially as described by the manufacturer. The Real-Time reaction was run under the following conditions: Segment 1: Initial denaturation 95C 10 minutes, Segment 2: 95°C 30 s, 55°C 1 min, 72°C 30 s, for 40 cycles, Segment 3: 95°C 1 min, ramp down to 50°C and ramp up from 50°C to 95°C. Primers amplifying *hrtB *(Per1-F + Per2-R), *hssR *(RR1-F+ RRS-R) and *ileS *(ileS-Forward + ileS-Reverse) which was used for normalisation: Per1-F:(5'TGAGGCACCTAAAATCGCTAC3'), Per2-R:(5'GGGAGAATATTTCGTTATTTGAACAC3'), RR1-F:(5'ACATTGATGCATACACACAACC3'), RR2-R:(5'GTCAACTGTTCGCTCATCTCC3'), ileS-Forward:(5'TTTAGGTGTTCGTGGTGA3'), ileS-Reverse:(5'CTTTATCTGCCATTTCTCC3'). All steps were repeated in three independent experiments giving similar results. Statistical analysis on QRTPCR results using GraphPad prism5, 1Way Anova with Dunnett's Multiple comparison test (GraphPad Software, Inc) determined changes in expression comparing time 0 to time 10 minutes or 90 minutes.

### Stress and antibiotic resistance of *S. aureus and L. monocytogenes*

Cultures of *S. aureus *and *L. monocytogenes *were grown exponentially in TSB and BHI, respectively, at 37°C. At an absorbance at 600 nm of 0.2 +/- 0.05 the cultures were diluted 10^-1^, 10^-2^, 10^-3 ^and 10^-4^fold, and 10 μl of each dilution was spotted on TSB or BHI plates. The plates were incubated at the indicated temperatures. In addition plates containing 4% NaCl were spotted and incubated in a similar way.

Antimicrobial susceptibility to ampicillin, gentamicin, sulfa/trimethoprim, rifampicin, tetracycline, amoxy/clavulan, cephalotin, clindamycin, enrofloxacin, fusidic acid and oxacillin was performed with a commercially available MIC technique using dehydrated antimicrobials in microtitre wells (Trek Diagnostic Systems Ltd., UK).

## Authors' contributions

LET participated in the design of the study, did the *S. aureus *transposon mutant library, growth- and complementation analysis, stress and antibiotic analysis, northern blot, transduction, extracellular protein analysis, *in vitro *killing assay and drafted the manuscript, CTG did the *L. monocytogenes *transposon mutant library, carried out the screening, MIC determinations and ATP leakage analysis, participated in the design of the study and helped revise the manuscript. SG did complementation, QRT-PCR, growth experiments with and without plectasin and hemin and DNA binding analysis. TTW screened the *S. aureus *transposon library and identified the *hssR *gene. HHK supplied the peptides, plectasin, eurocin, novicidin, and novispirin G10. LG and HI participated in the design of the study and helped revise the manuscript. All authors read and approved the final manuscript.
